# Newborn Aides: An Innovative Approach in Sick Newborn Care at a District-level Special Care Unit

**Published:** 2007-12

**Authors:** Amitava Sen, Dilip Mahalanabis, Arun K. Singh, Tapas K. Som, Sudipta Bandyopadhyay, Sutirtha Roy

**Affiliations:** 1 Society for Applied Studies, CF-198, Salt Lake, Sector 1, Kolkata 700 064, India; 2 Department of Neonatology, Institute of Post Graduate Medical Education and Research, Kolkata 700 020, India; 3 Newborn Unit, District Hospital, Purulia 723 101, India

**Keywords:** Cost-benefit analysis, Delivery of healthcare, Newborn care, Infant mortality, Nurses', Aides, Training, India

## Abstract

A Sick Newborn Care Unit (SNCU), established in a district hospital in India, substantially reduced the neonatal mortality rate in the district; it, however, suffered from a dearth of trained nurses. Local girls with 10-12 years of school education underwent structured and hands-on training for six months, followed by a six-month internship at the SNCU and were assigned to it as stipendiary ‘Newborn Aides’. Based on the results of formal examinations, internal on-the-job assessment and interview of doctors, nurses, and parents and their technical skills and motivation were rated very high. Although the incremental cost of training is small, the cost of sustaining them, i.e. stipend and replacing attrition, needs to be addressed. Trained Newborn Aides may substantially alleviate human-resource constraint for SNCUs and Sick Newborn Stabilization units in smaller peripheral hospitals for care of sick newborns at an affordable cost.

## INTRODUCTION

An estimated 130 million babies are born each year globally, and about four million of them die in the neonatal period (1). A quarter of the global neonatal deaths occur in India (1). The infant mortality rate in India has remained almost unchanged since the early nineties, and the near-static rate of neonatal mortality, despite introducing several primary care-based strategies and programmes at the national level during that period, is considered to be the major reason for this (2). In India as a whole and in the state of West Bengal, neonatal deaths remained high at 62.4% and 58.7% of infant deaths respectively (3). Even after intensive state-level essential newborn-care training and operationalization programme in West Bengal, the neonatal mortality rate has not improved (4). We had reasons to believe that reduction of maternal mortality rate requires facilities for cesarean section and blood transfusion (emergency obstetric care), in addition to antenatal care and care at birth. Effective and accelerated reduction in neonatal mortality rate also needs back-up support of state-of-the-art Sick Newborn Care Units (SNCUs) in hospitals with a large number of deliveries. In Purulia, a poor and underdeveloped district of West Bengal with a high neonatal mortality rate, an SNCU was developed in the district hospital in September 2003 and is run by relocated staff from within the district. Approximately 6,000 deliveries take place in a year in this hospital. Results, in the form of number of lives saved and the estimated reduction in neonatal mortality rate for the whole district, even with a limited number of beds and staff, were so encouraging (5–7) that this model was put to scale by the state government in several other districts and by United Nations Children's Fund (UNICEF) in Port Blair, Andaman and Nicobar Islands.

The SNCU facilities provided controlled environment, individual warming and close monitoring devices, intravenous fluid and medications by infusion pump, central oxygen, oxygen generators, bedside procedures, e.g. resuscitation and exchange transfusion, portable x-ray, and in-house side laboratory services. It, however, did not include mechanical ventilation and specialized neonatal surgery.

One important constraint of ‘The Purulia Model’, as it is now known, was the severe shortage of nursing staff. Precious few were made available by moving them from other wards to the nursing-intensive sick newborn care. Separate posts were neither created nor sanctioned for the SNCU by the state government mainly because of administrative and budgetary constraints that have prevented new recruitments. Anticipating such a problem, one (DM) of the investigators proposed the concept of Newborn Aides to assist trained nurses, as an innovative approach in sick newborn care during the inauguration of the SNCU at the district hospital in Purulia.

### Newborn Aides

The aim of this initiative was to create a group of specially-trained young female volunteers (whom we called ‘Newborn Aides’). These volunteers were drawn from among a stable local population. It was expected that they would be able to share or take up the burden of activities relating to simple house-keeping of a sick newborn unit and basic care of normal or sick neonates under the guidance and supervision of the nursing staff in a health facility so that the trained nurses can concentrate on more intensive patient-care work. The objective was to release time of trained nurses for critical care. We expected that, with intensive training, they would even be able to deliver improved newborn-care services at the First Referral Units and make home-visits for sick neonates in the community.

The primary objective of this paper is to describe the process of implementation of this programme of Newborn Aides in reasonable details so that others in similar resource-constrained situations can replicate this work. We have also calculated the direct incremental cost of training. This description, we believe, is relevant for translating known technology for reducing the rate of neonatal mortality in developing countries.

## MATERIALS AND METHODS

### The selection process

Nine candidates per batch were selected to avoid crowding. We developed criteria for the selection of Newborn Aides. We felt that they should be from the same locality (within 3-km radius of the health facility), in the age-group of 18-25 years, have educational level of Class X school certificate, fluent in local language, preferably unmarried or widow, and able to devote at least one nursing shift of 8-12 hours per day in the health facility and show active interest and devotion to the training and the work. Restricting the recruitment to recent school-leavers who are young and are less likely to be already burdened with many family and social commitments was based on our judgement that they are more likely to be able to devote long hours of duty. For similar reasons, unmarried or widowed women were preferred.

For the first batch (2005–2006), we arranged for the Purulia district branch of Indian Red Cross Society, to select seven of the nine candidates from Purulia town for the SNCU at Purulia, and the Panchayat Samity (local self-government) Manbazar block selected two with an assurance that they would beused in their Sick Newborn Stabilization Unit (SNSU) in future. One candidate from Purulia did not want to continue and was replaced. The second batch of nine (2005-2006) consisted of three candidates, each selected by the Panchayat Samity at Balarampur, Kotshila, and Hura blocks of Purulia district with an assurance that they would be used in their hospitals for the SNSUs in future. Two candidates did not want to continue and were replaced. The local Panchayat Samity did not fill up one remaining post for Manbazar. The Panchayat Samities did not always strictly follow the selection criteria. Three were overqualified (graduates), one was aged 35 years, and several in the second batch were staying at a distance of more than three km from the respective health facilities. Details of the 18 selected candidates (two batches) are presented in Table 1.

**Table 1:** Age, education, work experience, and marital status of selected candidates from batches of newborn aides (n=18)∗

Characteristics	Number
Mean age	21 (range 17-35) years
Educational level	Twelve years school leaving certificate : 3 Ten years school leaving certificate : 12 Graduate Graduate : 3
Past experience as health worker	One candidate only
Marital status	Unmarried : 11 Married : 6 Widow : 1
Number and reasons for drop-out	Batch I: 2 (one for poor performance and refusal to take additional period of training. Another left after 3 days without stating any reason) Batch II: 2 (local self-government changed both candidates within 7 days without showing any reason)

∗Seventeen of 18 candidates in two batches were certified after completion of internship

### The training programme

No tailored and readily-available curriculum was available for the above-mentioned training. The team (AS, DM, and AKS) developed a need-based curriculum consisting of six months of training, followed by six months of internship. The trainees were given a stipend of Rs 600 (US$ 14) only per month during training and Rs 800 (US$ 18) only per month during internship. The out-station candidates were provided with simple accommodation in the hospital premises. The training programme was initiated by a welcome meeting with the departmental staff, district (Panchayat, general, and health) administration, district Red Cross, and representatives from the Department of Neonatology, Institute of Post Graduate Medical Education and Research, and The Society for Applied Studies. The candidates were given two sets of uniform, an identification tag, and a set of basic handouts in vernacular. The training consisted of a series of introductory talks by the medical (SB and SR) and nursing-staff and acclimatization in the sick newborn-care unit, paediatric ward, labour room, and postnatal wards for two weeks. The introductory talks included: (a) newborn-related definitions; (b) cleanliness and disinfection; (c) care of normal newborn at birth; (d) care of newborn who did not cry at birth; (e) breastfeeding; (f) care of low birthweight (LBW) newborn; (g) keeping baby warm, including Kangaroo Mother Care (KMC); (h) danger signs; (i) referral and transport; and (j) use and maintenance of common equipment and were taught in multiple classes which were prepared from standard medical and nursing neonatology textbooks. The SNCU at this district hospital and its impact on mortality have been described earlier (5–7).

This was followed by rotational shift duty with a trained nursing staff for each trainee to closely observe her protocols and activities. After gaining some confidence, they were allowed to perform simple functions, e.g. cleaning of equipment, cleaning and changing nappies, etc. With time they took part in more and more sophisticated level of sick newborn care under supervision. The medical officers of the unit took them on rounds with the nurse on duty. They continued to take topic-oriented discussions and question-answer sessions on specified days. The trainees were also taken to visit the established SNSUs at the Block Primary Health Centres (BPHCs) and/or rural hospitals (Community Health Centres) for work experience. This on-the-job component of their training was considered to be the most important part of the training.

### The evaluation process

The trainees were under strict surveillance for internal assessment by the three medical officers and five senior nursing-staff. They individually gave marks after each fortnight, and the marks were averaged for a final score. Total 35 marks were based on a five-point scale for each of the following seven preset criteria: (a) punctuality and regularity; (b) discipline; (c) behaviour with trainers; (d) behaviour with parents; (e) knowledge and capacity to learn; (f) work culture and aptitude; and (g) willingness for taking additional responsibility. The averages of the marks obtained from the internal assessments on these criteria were added to the mid-term and final evaluations. Three external examiners (senior neonatal nurses and nurse-teachers) also evaluated the candidates in two examinations at three and six months before final and satisfactory certification. The three external examiners assessed each candidate, including hands-on-demonstration covering the priority areas and scored them independently. The average of the three was recorded. The distribution of marks out of 100 was as follows:

Internal assessment (mean of internal assessors)-35Picture spot (5 items identification and question)-5Oral (10 preset questions, 3 marks each)-30Practical (6 items, 5 marks each)-30

The photographs for identification covered the danger signs and the signs for identification of preterm and small-for-date neonates. These were from the collection of one (AS) of the authors, and the trainees were not earlier exposed to those photographs.

External assessors (a public-health expert with MD in Preventive and Social Medicine from UNICEF and Nurse-Tutors) were invited to assess the examination independently and give their opinions in writing.

Qualitative evaluation was also made through interview and group discussions with the concerned doctors, nurses, and Newborn Aides, also through assessing the comments of parents about their impression of the service by the Newborn Aides.

### Period of internship

During the internship, after completing the six-month training, the Newborn Aides were rotated through three areas in the district hospital where the neonates are located—the labour room and the postnatal ward, the paediatric ward, and the SNCU—in batches of 2-3 for two months in each area under guidance and supervision of the nursing-staff and medical officers. The internal assessment also continued during the six-month internship, and deficiency, if any, was discussed with them individually. Three doctors and five nurses evaluated the performance of the interns on a standard format once a month independently of each other.

## RESULTS

### Hurdles to implementation

The nursing association and nursing administration raised strong objections against the new concept of Newborn Aides because of the mistaken belief that this would take away nursing jobs from trained nurses. Initially, it was also difficult to train them in basic newborn resuscitation in the labour room because of these objections. The SNCU was operational for about one and a half years with an extreme shortage of nursing-staff before the nursing association and nursing administration could be persuaded to agree.

### Examination results

All participants, except one, scored high in the identification of the unseen photographs on the basis of their actual exposure to similar clinical conditions during training. This has validated their ability to identify a small neonate and the potential danger signs.

In the pooled internal assessments scores, their technical skills and motivation were rated as exceptionally good (results are not shown separately).

The examiners rated them ‘good’ to ‘excellent’, except one. The results of the final examinations of the two batches are as follows:

**Table:** 

Grade A+ (80% and above)	-	1
Grade A (70-79%)	-	6
Grade B (60-69%)	-	10
Grade C (below 60%)	-	1

The one with Grade C was asked to continue training for two more months, which she declined and left. Incidentally, she was a graduate and had other options left to her.

The external assessors opined that the Newborn Aides have proved themselves to be effective newborn caregivers under guidance and supervision in different hospital situations, i.e. at SNCU and SNSU.

### Internship

The tasks carried out by the Newborn Aides at the SNCU included: help the nursing-staff maintain asepsis, keep baby clean, train mothers, and assist the staff nurses with all the procedures they do. Their job included Kangaroo Mother Care, bag and mask procedure (under supervision), and assuring that the babies are put onto the breast soon after birth. Based on internal assessment, the authors found that they were empowered to carry out many essential newborn-care activities and, at the same time, perform state-of-the-art care of the sick neonates. Two of the authors evaluated the results of internal assessment and found that knowledge and practice were rated as ‘good’ to ‘excellent’. However, no formal examination was taken at the end of internship.

The rotation in the labour room and postnatal wards had a visible impact on initiating breastfeeding (within half an hour after delivery), teaching mothers how to keep their babies warm (often doing it themselves as they had hands-on-training), and taking part in early detection and referral (by bringing to notice of nurses and doctors) of the sick neonates. The perceived impact was based on internal assessment and open-ended interview of the newborn aides, concerned staff nurses, and the doctors at the SNCU.

Based on the group discussions and interview of the doctors and nurses at the SNCU, we found that the newborn aides have created and sustained a definite positive impact on neonatal care by augmenting the day-to-day newborn-related basic activities in the hospital, thereby reducing the workload of the insufficient number of relocated nursing staff made available for facility-based sick newborn care.

### Cost of training Newborn Aides

In an established and functioning SNCU, a necessary requirement for training, the cost of training for a batch of newborn aides was small and appeared to be cost-effective (Table 2). However, a formal cost-effective analysis was not conducted. The maximum number of newborn aides in a batch should not be more than the number of trained regular nurses posted in that SNCU. In calculating the cost, we did not include the establishment and running costs of the SNCU, including the salary of regular staff. The cost would also vary depending on the residential status of the trainees, local or out-station (as accommodation would be needed), and the rate of stipend.

**Table 2:** Cost of training a batch of 10 newborn aides based on Purulia experience

Item	Cost (Rs)
Selection process at local level	5,000 (one time)
Stipend during training @ Rs 800 per head per month	48,000 (for six months)
Two sets of uniform per head @ Rs 250 each	5,000 (one time)
Training materials @ Rs 1,000 per head	10,000 (one time)
Additional remuneration to local trainers	
For 3 MOs @ Rs 1,500 per head per month	27,000 (for six months)
For 10 nurses @ Rs 1,000 per head per month	60,000 (for six months)
Two tours to Sick Newborn Stabilization units	10,000
Mid-term and final examinations, including honorarium, travel, and daily subsistence cost of four examiners @ Rs 2,500 per head per examination	20,000
Stipend during internship @ Rs 1,000 per head per month	60,000 (for six months)
Certifcation ceremony and prize distribution	5,000 (one time)
Organizational cost, including supervision of training by implementing agency and consultancy honorarium of programme coordinator (neonatologist)	80,000 (for one year)
Total	330,000 per batch of 10 (Approximately US$ 7,334)

MOs=Medical Officers

The direct cost of training for a batch of 10 newborn aides in a functioning district-level SNCU with 10 trained nurses and three medical officers as trainers has been estimated to be Rs 330,000 only or Rs 33,000 (US$ 733) only per trainee till certification at the end of one year (Table 2).

The minimum cost of using each newborn aide after certification is expected to be Rs 1,200 per month, i.e. Rs 14,400 (US$ 320) only per year. It is meant to be an incentive, not a salary. It is anticipated that a significant number will take up jobs at private medical establishments or will out-migrate. This will increase the cost because more will need to be trained. The Newborn Aides working in the SNSUs would need some short refresher course in the SNCU of the district every year or two. The cost of such refresher courses has not been included.

## DISCUSSION

While intervention programmes for reducing the neonatal mortality rate should start with family and community care and outreach services, strengthening clinical service, a much-needed component, is largely ignored by health planners. The non-availability of adequate sick newborn care at fixed facilities, such as district hospitals and below, largely negates the value of early referral of sick neonates. Even where one is able to create facilities for quality care for sick neonates, the near-unsolvable problem of providing trained nurses by the healthcare system makes it difficult to sustain them.

SNCUs at level II and III, as defined by the National Neonatology Forum, India (8), often form a component of the paediatric departments of teaching hospitals in India. Our innovation was to successfully position such level II units in district/peripheral hospitals as a public-health tool for reducing the neonatal mortality rate in the district as a complement to essential newborn care. This SNCU concept was combined with smaller newborn-care units at peripheral hospitals for stabilization and a functional/affordable transport for sick neonates. This successful model, now popularly known as Purulia Model of sick newborn care, has been described and widely disseminated to health planners and policy-makers in India (5–7).

Our second innovation—the topic of this paper—was the development of the concept of newborn aides and its successful implementation in the context of a SNCU. This innovation addresses the problem of severe shortage of trained nurses for sick newborn care at fixed facilities, including at-first referral units. Young girls with 10-12 years of school education, residents of the community where the health facility (SNCU/SNSU, etc.) is located, when empowered as newborn aides with an intensive hands-on-training for six months, followed by internship for six months at an SNCU, can form a major force in effective reduction of neonatal mortality. No comparable reference is available in literature for such multifaceted use of newborn caregivers. The Policy Statement for Minimal Standards of Neonatal Care in Hospitals by the Pediatric Society of Papua New Guinea recommended the use of ‘Nurse Aides’ in 1:1 ratio with nurses for all levels of newborn care (9). However, other than mentioning the term ‘Nurse Aides’, no details of their training, evaluation, use, and job description have been stated.

Considering the requirement of at least three newborn aides for each SNSU (to cover 24 hours a day, 7 days a week) at rural hospitals and BPHCs and 10 newborn aides for the SNCU at the district hospital, a district with 15 SNSUs and one SNCU as an example, would require 55 trained newborn aides. In addition, 15 newborn aides would be required to cover an anticipated attrition rate of 25%. With an intake of 10 newborn aides every six months, four years would be required to train the necessary number for that district.

The newborn aides were found to be highly motivated and highly skilled in normal and sick newborn care. They are capable of forming the critical mass of trained personnel to sustain high-quality newborn care in the peripheral health facilities in the districts with minimal support. We can only speculate on motivation for attaining and sustaining excellence by the newborn aides. They may include high self-esteem and the attention of and recognition by doctors, nurses, and parents of sick babies and by society at large. Job satisfaction as stated and perceived by the staff members were rated ‘high’.

Training and evaluation of the newborn aides should get due recognition from the appropriate authority. Many other districts and states in India are looking forward to training such workers to reduce their high neonatal mortality rate and to solve the problem of a human-resource constraint for running SNCUs. We initiated steps to locate financial support for the newborn aides after they were trained, and the initial responses from the district administration were encouraging. Although the financial obligation for sustaining the trained newborn aides is small, their long-term sustainability remains presently to be addressed.

### Retention of Newborn Aides

Some structural problems need to be addressed before they could be effectively used which include (a) financial support for them, e.g. local adoption, job-based compensation, and (b) need for creating a support structure (enabling mechanism). Despite the highly-successful Purulia Model, the Panchayat system (local self-government) in Purulia has not yet shown sufficient interest in creating such a mechanism. It is not unlikely that some of these highly-trained women would move to private hospitals and institutions or to some other professions. A sustained support from the Government and the administration will go a long way in retaining these highly-skilled workers.

There are constraints to, and limitations of, this model of newborn aides for care of sick newborns. For the sustainability of these local-level trained workers, we need their adoption by the local self-government or other agencies. It also needs a sustained political will and support. For scaling up of this programme, the difficulty of providing external inputs at this level needs to be addressed. The attrition rate is difficult to foresee at this point. We expect out-migration due to multiple reasons, including marriage, higher-education opportunities, and better job offers. The latter can partly be addressed by both financial and non-financial incentives. It should also be noted that the programme is based on intensive on-the-job training, and its success depends on the quality of SNCU and their nursing and medical staff.

In the light of our earlier experience with the SNCUs as a tool for reducing the neonatal mortality rate (5–7) and with newborn aides as an innovative solution for nursing shortages, it is pertinent to comment on some policy issues. Health-intervention strategies are not meaningful without a mechanism for delivery; both effectiveness and cost are dependent on this. Development of health systems should be considered a phased process, starting with the use of existing institutions and available resources—both physical and human. For sustainable development, new healthcare interventions should be implemented through the existing healthcare systems and, with time, should be extended by adding and deepening the network of services (10). While health interventions in isolation are occasionally helpful, they work best when integrated with the existing system.

A textbook for newborn aides in Bangla has now been developed and is undergoing field-testing.
